# Polymorphism of *UBC9* Gene Encoding the SUMO-E2-Conjugating Enzyme and Breast Cancer Risk

**DOI:** 10.1007/s12253-013-9659-4

**Published:** 2013-07-20

**Authors:** Katarzyna Wozniak, Renata Krupa, Ewelina Synowiec, Zbigniew Morawiec

**Affiliations:** 1 Department of Molecular Genetics, Faculty of Biology and Environmental Protection, University of Lodz, Lodz, Poland; 2 Department of Surgical Oncology, N. Copernicus Hospital, Lodz, Poland

**Keywords:** UBC9 gene and protein, Breast cancer, Sumoylation

## Abstract

UBC9 protein (E2-conjugating enzyme) plays a key role in post-translation modification named sumoylation. Proteins, which are sumoylated take part in many cellular processes including cell growth, maintaining the genome integrity and stability and cancer development. The aim of this study was to investigate an association between three polymorphisms of the *UBC9* gene: c.73G>A (rs11553473), c.430T>G (rs75020906) and g.1289209T>C (rs7187167) and a risk of ductal breast cancer occurrence. We performed a case-control study in 181 breast cancer cases and 277 controls using PCR-RLFP and ASO-PCR. In the case of the 430T>G polymorphism of the *UBC9* gene lack of variability suggests that there is not a polymorphic site in polish population. We observed that a risk of breast cancer occurrence is elevated in patients with the G/A genotype (OR 5.03; 95 % Cl 3.05–8.28), the A/A genotype (OR 11.3; 95 % Cl 4.24–30.3) and the A allele (OR 6.86; 95 % Cl 4.43–10.6) of the c.73G>A polymorphism. In the case of the g.1289209T>C polymorphism we found a correlation between estrogen receptor (ER) expression and the T/T genotype (OR 0.22; 95 % Cl 0.07–0.64) and the T allele (OR 0.53; 95 % Cl 0.32–0.88). We also found a correlation between the T/T genotype (OR 4.13; 95 % Cl 1.21–14.1) and the T allele (OR 2.09; 95 % Cl 1.07–4.08) of the g.1289209T>C polymorphism with triple negative breast cancer. Our results suggest that the variability of the *UBC9* gene can play a role in breast cancer occurrence.

## Introduction

Small ubiquitin-related modifier (SUMO-1, 2 and 3) conjugation is a type of post-translational modification of proteins named sumoylation. Sumoylation is the process that requires few steps of enzymatic reactions, like maturation, activation, conjugation and ligation. Mature SUMO protein is activated by the heterodimeric SUMO E1 enzyme (SAE1/SAE2). Next, SUMO protein is displaced from the E1 complex to the E2 conjugating enzyme named UBC9. The enzyme catalyzes the formation of izopeptide bond between the C-terminal Gly residue of SUMO protein and ɛ-amino group of Lys residue in the target protein. In the last step SUMO protein is attached to its substrate. This step can occur on two ways: directly by E2 or by the E3 enzyme [1]. Sumoylation is similar to ubiquitination, but the biological functions of these two processes can be quite distinct. Unlike ubiquitination that normally targets proteins for degradation through proteasome pathway, sumoylation regulates divers cellular processes, including DNA replication and repair, chromosome packing and dynamics, genome integrity, nuclear transport, signal transduction and cell proliferation [2].

UBC9 is the only known E2 conjugating enzyme that exists for sumoylation. It exerts a central function for the sumoylation pathway, interacting with almost all the partners required for sumoylation. In addition, recent evidences indicate that UBC9 is a multifunctional protein that can exert its functions independent of sumoylation [3, 4]. It is over-expressed in several cancers like colon, prostate, breast, lung, ovarian, melanomas, head and neck [5–7]. Wu et al. [8] reported that in breast cancer the UBC9 level was >5-fold higher than the matched normal tissues. UBC9 has also been shown to bind to HMG1 proteins and integrate both positive and negative signals for proliferation and transformation [9]. Recently UBC9 was found to promote cell invasion and metastasis of breast cancer cells [4], implicating a role in tumorigenesis.

SUMO modification can influence a plethora of transcription factors and co-factors. Sumoylation of transcription factors can lead to transcriptional activation but has been mostly associated with transcriptional repression. Attenuation or repression of their transcriptional activity has been observed for the majority of nuclear receptors like ER (estrogen receptor), PR (progesterone receptor) and AR (androgen receptor) [10–12].

The SUMO modification pathway was also shown to be involved in response to DNA damage [13, 14]. Number of studies suggests a link between repair of DNA, especially DNA double strand breaks (DSBs) and genetics predisposition to hereditary as well as sporadic breast cancer [15–18]. Known breast cancer susceptibility genes like *BRCA1, BRCA2, ATM, CHK2, TP53* are involved in the repair of DSBs and related processes such as cell cycle control, indicating that disturbances of DNA DSBs repair might result in breast cancer development.

UBC9 protein, the key enzyme of sumoylation, may take part in breast cancer development resulting in a change of localization, activity and stability of modified proteins. For this reason, UBC9 may also serve as a potential biomarker for diagnosis or prognosis as well as a therapeutic target for breast cancer therapy. Genetic variability may affect expression and activity of UBC9 gene or/and protein and may have an impact on breast cancer occurrence and progression.

In the present study, we investigated a correlation between three polymorphic variants (SNPs) of the *UBC9* gene (c.73G>A rs11553473, c.430T>G rs75020906, g.1289209T>C rs7187167) and breast cancer risk. We also studied an association between the above polymorphisms of the *UBC9* gene and clinical characteristics of breast cancer patients such as tumor grade, tumor stage and ER and PR (estrogen and progesterone receptors, respectively) and HER2 expression (human epidermal growth factor 2).

## Patients, Materials and Methods

### Patients

Blood samples were collected from 181 women (mean age 60 ± 11 years) diagnosed with ductal breast cancer treated at the Department of Surgical Oncology, N. Copernicus Hospital (Lodz, Poland). Blood was collected before surgical treatment and chemotherapy. The control group consisting of 277 women is age-matched women who were not diagnosed with cancer and recruited from Commune Health Clinic in Rzgow and Institute Polish Mother’s Health Center (Lodz, Poland). The Local Ethic Committee approved the study and each patient gave a written consent.

### Genomic DNA Isolation

Genomic DNA was prepared from peripheral blood of breast cancer patients and healthy individuals by using of commercial Blood Genomic DNA Miniprep Kit (Axygen Biosciences, CA, USA), as recommended by the manufacturer.

### Selection of Polymorphisms and Primers Design

We obtained a list of SNPs in the *UBC9* gene from the public domain of the National Center for Biotechnology Information–the Single Nucleotide Polymorphisms database (NCBI dbSNP) at http://www.ncbi.nlm.nih.gov/snp. For this study we chose the three polymorphisms of the *UBC9* gene: c.73G>A (rs11553473), c.430T>G (rs75020906) and g.1289209T>C (rs7187167) with minor allele frequency higher than the 1 %. Primers were designed using Primer–Blast software (http://www.ncbi.nlm.nih.gov/tools/primer-blast/) and Web-based allele-specific primer software in the case of the c.73G>A (rs11553473) (http://bioinfo.biotec.or.th/WASP).

### Genotype Determination

The restriction fragment length polymorphism reaction (PCR-RFLP) was used to determine the genotypes of the c.430T>G and the g.1289209T>C polymorphisms of the *UBC9* gene. The allele-specific polymerase chain reaction (ASO-PCR) was used to determine the genotypes of the c.73G>A polymorphism of the *UBC9* gene.

PCR reaction was performed in a total reaction volume of 25 μl containing 50 ng of genomic DNA, 1 U Biotools DNA polymerase (Biotools, Madrid, Spain), 1 × reaction buffer (750 mM Tris–HCl (pH 9.0), 500 mM KCl, 200 mM (NH_4_)_2_SO_4_), 1.5 mM MgCl_2_, 0.2 mM of each dNTP and 0.25 μM of each primer (Metabion, Martinsried, Germany and SIGMA-ALDRICH Co. St. Louis, MO, USA). PCR amplifications were conducted in DNA Engine thermal cycler (Bio-Rad Laboratories, Hercules, CA, USA). Thermal cycling conditions were as follows: initial denaturation step at 95 °C for 5 min, 34 cycles at 95 °C for 30 s, 30 s at 63 °C, 63.5 °C and 76 °C annealing temperature for c.73G>A, c.430T>G and g.1289209T>C polymorphisms respectively, elongation step at 72 °C for 60 s and final elongation step at 72 °C for 5 min.

The products of the c.430T>G polymorphism of the *UBC9* gene were digested 1 h with 0.2 U of the restriction enzyme *Rsa*I and the products of the g.1289209T>C polymorphism of the *UBC9* gene were digested 2 h with 0.2 U of the restriction enzyme *Bsa*HI (NEB New England Biolabs, Ipswich, MA, USA). The PCR products of the g.1289209T>C and the c.430T>G polymorphisms were separated into 10 % polyacrylamide gel and the PCR products of the c.73G>A polymorphism were separated onto 3 % agarose gel, stained with ethidium bromide and viewed under UV light.

### Statistical Analysis

Statistical analysis was performed using Sigma Plot 11.0 statistical package. A linkage between genotype, cancer and clinical parameters was accessed by the logistic regression. In all tests *p* values of less than 0.05 were considered statistically significant.

## Results

### Genotype Analysis

Breast cancer patients and controls were divided into groups corresponding to three genotypes. In the case of the c.430T>G polymorphism of the *UBC9* gene we observed only the T/T genotype. Therefore, above place cannot be treated as a polymorphic site (data not shown). The distribution of genotypes of the c.73G>A and the g.1289209T>C polymorphisms of the *UBC9* gene for cancer patients and controls is shown in Table 1. We observed a strong association between breast cancer occurrence and the G/A genotype (OR 5.03; 95 % Cl 3.05–8.28), A/A genotype (OR 11.3; 95 % Cl 4.24–30.3) and A allele (OR 6.86; 95 % Cl 4.43–10.6) of the c.73G>A polymorphism of the *UBC9* gene. We also observed a strong correlation between cancer and combination of genotypes: GA/CC, AA/CC and AA/CT. Protective effect was observed for combination of GG/CT genotypes of the c.73G>A and g.1289209T>C polymorphisms.

**Table 1:** The genotype and allele distribution and odds ratios (OR) of the g.1289209T>C and the c.73G>A polymorphisms of the *UBC9* gene in breast cancer patients and controls

Genotype or allele	Breast cancer patients (*n* = 181)	Controls (*n* = 277)	OR	OR (95 % Cl)	*P value*
g.1289209T>C					
C/C	85	120	*1.00 ref.*		
C/T	80	148	0.90	0.58–1.38	0.615
T/T	16	7	2.58	1.00–6.65	0.050
C	250	388	*1.00 ref.*		
T	112	162	1.24	0.87–1.77	0.232
c.73G>A					
G/G	52	228	*1.00 ref.*		
G/A	89	40	5.03	3.05–8.28	<0.001
A/A	40	7	11.3	4.24–30.3	<0.001
G	193	496	*1.00 ref.*		
A	169	54	6.86	4.43–10.6	<0.001
c.73G>A/g.1289209T>C					
GG/CC	22	102	*1.00 ref.*		
GG/CT	25	119	0.116	0.063–0.211	<0.001
GG/TT	5	7	0.471	0.135–1.639	0.236
GA/CC	41	15	3.456	1.64–7.283	0.001
GA/CT	41	25	1.439	0.771–2.689	0.253
GA/TT	7	0	5.704	0.671–48.488	0.111
AA/CC	22	3	5.227	1.464–18.667	0.011
AA/CT	14	4	7.47	1.58–35.31	0.011
AA/TT	4	0	4.686	0.391–56.184	0.223

There was no difference in the frequency of the genotypes and alleles of the g.1289209T>C polymorphism of the *UBC9* gene between patients and controls.

### Hormone Receptor Status

We checked the distribution of genotypes and alleles of the *UBC9* gene polymorphisms in groups of patients with different hormone receptor status. An association between estrogen hormone receptor status and the T/T genotype (OR 0.22; 95 % Cl 0.07–0.64) and T allele (OR 0.53; 95 % Cl 0.32–0.88) of the g.1289209T>C polymorphism of the *UBC9* gene was found (Table 2). We did not observe any association between estrogen hormone receptor status and the distribution of genotypes and alleles for the c.73G>A polymorphism of the *UBC9* gene (data not shown). We did not observe any association between progesterone hormone receptor status, HER2 expression and the distribution of genotypes and alleles for analyzed polymorphisms of the *UBC9* gene (data not shown). We found a correlation between the T/T (OR 4.13; 95 % Cl 1.21–14.1) and T allele (OR 2.09; 95 % Cl 1.07–4.08) of the g.1289209T>C polymorphism of the *UBC9* gene and the triple negative breast cancer (Table 3).

**Table 2:** The genotype and allele distribution and odds ratios (OR) of the g.1289209T>C and c.73G>A polymorphisms of the *UBC9* gene in groups of patients with different estrogen receptor status

Genotype or allele	ER positive (*n* = 119)	ER negative (*n* = 53)	OR	OR (95 % Cl)	*P value*
g.1289209T>C					
C/C	62	21	*1.00 ref.*		
C/T	51	22	1.07	0.55–2.08	0.852
T/T	6	10	0.22	0.07–0.64	0.006
C	175	64	*1.00 ref.*		
T	63	42	0.53	0.32–0.88	0.014
c.73G>A					
GG	35	14	*1.00 ref.*		
GA	58	27	0.90	0.47–1.74	0.757
AA	26	12	0.95	0.43–2.09	0.903
G	128	55	*1.00 ref.*		
A	110	51	0.95	0.43–2.09	0.903

**Table 3:** The genotype and allele distribution and odds ratios (OR) of the g.1289209T>C and c.73G>A polymorphisms of the *UBC9* gene for triple negative breast cancer patients

Genotype or allele	Triple negative (*n* = 24)	Others (*n* = 142)	OR	OR (95 % Cl)	*P value*
g.1289209T>C					
C/C	8	72	*1.00 ref.*		
C/T	11	60	1.07	0.43–2.63	0.886
T/T	5	10	4.13	1.21–14.1	0.024
C	27	204	*1.00 ref.*		
T	21	80	2.09	1.07–4.08	0.030
c.73G>A					
GG	8	40	*1.00 ref.*		
GA	11	71	0.89	0.37–2.17	0.796
AA	5	31	0.96	0.33–2.85	0.944
G	27	151	*1.00 ref.*		
A	21	133	0.92	0.49–1.72	0.793

Triple negative–ER, PR and HER2 negative breast cancer patients

Others–at last one receptor positive breast cancer patients (ER or PR or HER2 positive)

### Clinical-Histopathological Parameters

We checked the distribution of genotypes and alleles of the *UBC9* gene polymorphisms for breast cancer patients stratified by Bloom-Richardson grading system and TNM staging. We did not find any association between the c.73G>A and the g.1289209T>C polymorphisms of the *UBC9* gene and Bloom-Richardson tumor grade and TNM stage (data not shown).

## Discussion

In this study, we analyzed an association of the three polymorphisms of the *UBC9* gene: c.73G>A (rs11553473), c.430T>G (rs75020906) and g.1289209T>C (rs7187167) with a risk of ductal breast cancer occurrence. We observed a strong association between breast cancer occurrence and the G/A and A/A genotypes and the A allele of the c.73G>A polymorphism (Table 1). These genotypes and the A allele increased a risk of breast cancer occurrence. Moreover, we found that the some genotype combinations of the g.1289209T>C and c.73G>A polymorphisms correlated with breast cancer risk (Table 1). We have not noted variation in the frequency of genotypes associated with the c.430T>G (rs75020906)-*UBC9* gene polymorphism (data not shown).

The most interesting results we obtained by analyze the g.1289209T>C polymorphism and receptors status. We found that the variant allele was inversely related to ER positive breast cancer (Table 2). What is more, we observed that the T/T genotype and the T allele positively correlated with phenotype of triple negative breast cancer (Table 3). This is a subtype of breast cancer characterizing by the lack of expression of ER, PR and HER2 and by this way is called a triple negative breast cancer (TNBC). This subgroup accounts for about 15 % of all breast cancers and for a higher percentage of breast cancers detected in African and African-American women who are premenopausal [19]. TNBC has important clinical implications, because it is typicality high grade, has a ductal histology and exhibits a high rate of proliferation. In general, compared with other subtypes of breast cancer, TNBC has a less favorable clinical outcome in terms of the nature and likelihood of progression, availability of various treatment options, and survival [20–22].

TNBC tumors are strongly associated with germline mutations in the *BRCA1* gene [20–22]. In sporadic breast cancer, BRCA1 mutations are rare, but reduced expression or aberrant subcellular localization of BRCA1 is common among young African-American women with TNBC [19]. BRCA1 is a tumor suppressor that undergoes active nuclear import and export, which can provide a regulatory function. Moreover, BRCA1 is a key mediator of DNA repair pathways, especially of DNA DSBs repair, and participates in the maintenance of the genomic integrity of cells [22]. The nuclear-cytoplasm shuttling of BRCA1 may offer an important mechanism for regulating its function. Nuclear transport of proteins can be altered either by mutations or through post-translational modifications include phosphorylation, ubiquitylation, glycosylation and sumoylation within or proximal to nuclear import/export signals which could cause a conformational change, thus preventing the binding of proteins like UBC9 resulting in non-nuclear distribution of BRCA1 proteins [19]. The results obtained by Qin et al. [19] and previously by Xu et al. [23] are consistent with the model that a direct interaction of BRCA1 with UBC9 is critical for growth-tumor suppression by BRCA1 and ER-α levels causing transformation. These results suggest a molecular interplay between BRCA1 and UBC9, which maintains the balance of two opposing effects: tumor suppression (inhibition of ER-α) or tumorigenesis (activating ER-α). BRCA1 dysfunction can tilt this delicate balance resulting in ER-α positive or ER-α negative breast cancer. BRCA1 thus functions as a master switch, which by turning off or on UBC9 binding regulates ER-α transcription and cell growth. This model explains how BRCA1 can both activate and repress ER-α transcription in hormone responsive tissues like breast and ovarian. Post-translational modification of BRCA1 proteins could prevent the binding of BRCA1 to UBC9 resulting in breast cancers [19].

There is little data on the genetic variation of the *UBC9* gene, its impact on the function of UBC9 protein and potential role in the development of breast cancer [24–26]. Previously, we showed that breast cancer patients with variant allele of the c.73G>A polymorphism of the *UBC9* gene (rs11553473) have decreased efficacy of DNA DSBs repair [26]. Further studies are needed to clarify the mechanism of DNA DSBs repair reduction observed in carriers of the variant allele.

In summary, our data suggest that genetic variants of the *UBC9* gene, c.73G>A (rs11553473) and g.1289209T>C (rs7187167), may play role in the development of breast cancer. Probably, these polymorphic variants cannot be considered as independent markers, but rather the elements of a set of breast cancer markers. Further studies are needed to clarify their biological functions during breast cancer development.
